# Genomic Characterization of the Kazakh Fat-Tailed Coarse-Wool Sheep Breed Using ROH Analysis

**DOI:** 10.3390/ani15182714

**Published:** 2025-09-16

**Authors:** Altynay Kozhakhmet, Zhanerke Akhatayeva, Kairat Dossybayev, Marina Yermekova, Tilek Kapassuly, Kanagat Yergali, Aibyn Torekhanov, Utepbergen Bissenov, Xianyong Lan, Beibit Kulataev

**Affiliations:** 1 LLP “Kazakh Research Institute for Livestock and Fodder Production”, Almaty 050035, Kazakhstan; altynaitg@gmail.com (A.K.); akhatayevazhanerke@163.com (Z.A.); nurii_90@mail.ru (M.Y.); tilek.kapas@mail.ru (T.K.); ergaly.qanagat@gmail.com (K.Y.); torehanov.aibyn@mail.ru (A.T.); bnar68@yandex.ru (B.K.); 2 RSE Institute of Genetics and Physiology SC MSHE RK, 93 Al-Farabi Avenue, Almaty 050060, Kazakhstan; 3 Faculty of Biology and Biotechnology, Al-Farabi Kazakh National University, Almaty 050040, Kazakhstan; 4 Institute of Grassland Research, Chinese Academy of Agricultural Sciences, Hohhot 010010, China; 5 Department of the Biology and Fish Farming, Kh. Dosmukhamedov Atyrau University, Atyrau 060011, Kazakhstan; bisenovy@mail.ru; 6 College of Animal Science and Technology, Northwest A&F University, Yangling, Xianyang 712100, China; lanxianyong79@126.com

**Keywords:** runs of homozygosity, sheep, inbreeding, SNP, F_ROH_, F_GRM_

## Abstract

**Simple Summary:**

Sheep are an important source of food and income for people in Kazakhstan. Among the local breeds, the Kazakh fat-tailed coarse-wool sheep is especially valued because it grows well on natural pastures, produces a large amount of meat, and can survive in very hot summers and cold winters. To keep this breed healthy and productive, it is important to understand its genetic background. In our study, we used 500 sheep to learn how much variety exists within the breed and to find genomic regions to underline useful traits. We discovered multiple genes that are connected to growth, fat storage, reproduction and wool production. This knowledge will help farmers and breeders improve their flocks while protecting the genetic resources that are so valuable for the future of sheep farming in Kazakhstan.

**Abstract:**

Sheep breeding is an important sector of livestock production in the Republic of Kazakhstan. The Kazakh fat-tailed coarse-wool sheep holds a prominent position among local breeds due to its high meat productivity, resilience to extreme climatic conditions, and efficient use of pasture resources. This study focuses on the analysis of runs of homozygosity (ROH) to evaluate the genetic diversity level, inbreeding and to detect selection signatures in the Kazakh fat-tailed coarse-wool sheep breed. A total of 500 animals were genotyped using the OvineSNP50 BeadChip (Illumina, San Diego, CA, USA). As a result, a total of 41,728 ROH segments were identified, with an average length of 1.59 Mb, distributed across the entire genome. The most prominent homozygous regions were detected on chromosomes OAR10, OAR13, and OAR22, which might be associated with selection signatures. Genomic inbreeding coefficients (F_ROH_ and F_GRM_) showed a strong positive correlation (*r* = 0.58, *p* < 0.001), supporting the effectiveness of ROH-based analysis. Several candidate genes were detected, including *MYF5*, *PRDM16*, *TGM3*, *SLC26A4* and *SMAD5* which are notably involved in muscle formation, wool traits, and fat metabolism. The findings have substantial practical value for breeding programs and for managing genetic diversity in sheep farming enterprises in the Republic of Kazakhstan.

## 1. Introduction

Sheep breeding is an important sector of livestock production in the Republic of Kazakhstan, providing the population with meat and wool. According to the Bureau of National Statistics under the Agency for Strategic Planning and Reforms of the Republic of Kazakhstan, the total sheep population in the country exceeded 22.6 million head in 2024 [1]. The Kazakh fat-tailed coarse-wool sheep holds a prominent position among local breeds due to its high meat productivity, resilience to extreme climatic conditions, and efficient use of pasture resources [2,3]. The Kazakh fat-tailed coarse-wool breed was recognized in 1994 [4] through complex reproductive crossbreeding of Kazakh fat-tailed sheep with Edilbay, Sarajin, Tajik, and Degeres rams. Distinctive features of the breed include a moderately sized fat tail, an optimal meat-to-bone ratio, and strong adaptation to desert and semi-desert environments. In light of the growing demand for sheep-derived products and the need for sustainable agricultural development, improving the genetic potential of meat breeds, applying modern selection methods, and monitoring genetic diversity in pasture populations have become increasingly important.

In this context, genomic evaluation tools, especially approaches based on runs of homozygosity (ROH), have gained considerable relevance. Today, ROH-based assessments are widely used to evaluate genetic diversity and inbreeding levels in livestock and to identify genomic regions that influence economically important traits [5,6,7,8]. ROHs are continuous homozygous stretches of the genome that are inherited from common ancestors in an identical-by-descent (IBD) state [9]. The number, length, and chromosomal location of ROH segments allow researchers to estimate inbreeding levels, reconstruct demographic history, and detect functionally important genomic regions associated with valuable traits [10,11]. The length of ROH segments indicates the timing of inbreeding events; long tracts (>10 Mb) point to recent consanguinity (within the past five generations), whereas medium (2–5 Mb) and short segments (<1 Mb) suggest more ancient common ancestry (up to 30 generations ago) [12,13,14]. The genomic inbreeding coefficient F_ROH_, calculated from ROH segments, reflects the proportion of the genome in an autozygous state. It is determined by dividing the total length of ROHs by the total autosomal genome length and provides a quantitative measure of individual inbreeding levels without relying on pedigree records [14,15,16]. An alternative way to estimate inbreeding is through the genomic inbreeding coefficient F_GRM_, based on the genomic relationship matrix (GRM). This approach evaluates the degree of inbreeding by measuring genotypic similarity between individuals and reflects the proportion of genomic variation due to shared ancestry [17].

The practical value of ROH-based evaluation has been demonstrated in various livestock species. In sheep, ROH islands have been linked to traits such as fertility, meat quality, and milk fat content [18,19,20,21]. For instance, ROH analysis using the Illumina OvineSNP50 BeadChip in African sheep breeds identified candidate genes such as *ANPEP*, *HDDC3*, *ST3GAL3*, and *GRK4* [20]. Selli et al. (2021) reported shared genomic regions of high homozygosity and heterozygosity across 17 global sheep populations [22]. Similar investigations in goats have shown the utility of F_ROH_ estimates and identification of homozygous regions associated with productive and adaptive traits in Chinese breeds [8,22,23]. In cattle, ROH exploration enables the estimation of inbreeding coefficients and the localization of regions containing genes that affect milk yield, body weight gain, and disease resistance [24,25].

The present study aimed to identify the level of inbreeding, ROH islands associated with productive traits, and functional annotation of the respective genes and biological pathways in Kazakh fat-tailed coarse-wool sheep. The results obtained may serve as a foundation for future breeding strategies aimed at improving the productivity of the Kazakh fat-tailed coarse-wool breed

## 2. Materials and Methods

### 2.1. Sample Collection

**Figure 1:**
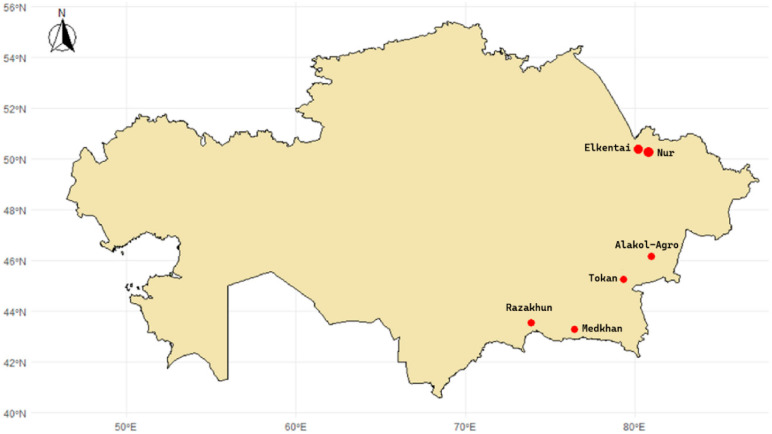
Map showing the geographical locations of the sampled farms in Kazakhstan.

A total of 500 Kazakh fat-tailed coarse-wool sheep were sampled from six farms in the southern and southeastern regions of Kazakhstan (Figure 1), namely Medkhan (43.29° N, 76.44° E), Razakhun (43.54° N, 73.86° E), Tokan-1 (45.27° N, 79.35° E), Alakol-Agro (46.17° N, 80.95° E), Nur (50.45° N, 80.29° E), and Elkentai (50.40° N, 80.20° E). Sampling was carried out during the weaning period, from 22 February 2023 to 22 February 2025.

Blood samples were collected via jugular venipuncture into EDTA-containing vacuum tubes and stored at −20 °C until transport to the laboratory.

### 2.2. DNA Extraction and Genotyping

DNA was extracted using the GeneJET Genomic DNA Purification Kit (Thermo Scientific, Waltham, MA, USA) following the manufacturer’s protocol. Concentration was measured on a NanoDrop 2000 spectrophotometer (Thermo Scientific, Waltham, MA, USA), with most values ranging between 50 and 70 ng/μL. To evaluate purity and check for possible degradation or contamination, selected samples were run on a 1% agarose gel.

SNP genotyping was performed using the OvineSNP50 Genotyping BeadChip (Illumina, San Diego, CA, USA). Samples were prepared according to the Infinium HD Assay Ultra Protocol using a Tecan robotic liquid handling system, and scanned on an iScan platform (Illumina, San Diego, CA, USA). Raw data were processed in GenomeStudio (v2.0.). SNPs with a call rate of at least 98% were retained during quality control. Final datasets were exported in PED and MAP file formats using the PLINK Input Report Plug-in.

Only autosomal SNPs were retained by excluding those located on sex chromosomes (X and Y) using bash script. Quality control (QC) for missing data was then performed in PLINK (V1.90) by following criteria: SNPs and individual samples with a missing genotype rate >5% were removed. After quality control, a total of 49,363 SNPs and 497 individuals successfully passed and were used for subsequent analyses. The number of individuals retained from each population was as follows: Tokan (n = 71), Nur (n = 98), Alakol-Agro (n = 65), Elkentai (n = 98), Razakhun (n = 86), and Medkhan (n = 79).

### 2.3. ROH Detection

Runs of homozygosity were identified using PLINK (v1.90) with the following threshold parameters: minimum SNP density within a ROH was set to at least 1 SNP per 100 kb (--homozyg-density 100), the maximum gap allowed between two consecutive SNPs within a ROH was 1000 kb (--homozyg-gap 1000), the minimum ROH length was 1000 kb (1 Mb) (--homozyg-kb 1000), and the minimum number of SNPs constituting a ROH was 15 (--homozyg-snp 15). Additionally, a maximum of one heterozygous SNP per window was allowed (--homozyg-window-het 1), and a sliding window size of 15 SNPs was used to scan the genome for ROHs (--homozyg-window-snp 15) [16].

### 2.4. Estimation of Genomic Inbreeding and Correlation Analysis

Genomic inbreeding was evaluated using two complementary metrics. The first, genomic relationship matrix (F_GRM_), was calculated in PLINK (v1.90) and GCTA (v1.94.1) using the --make-grm and --grm-inbreeding options, based on a genomic relationship matrix constructed from all autosomal SNPs. This coefficient reflects how much observed homozygosity deviates from what would be expected under random mating. The second metric, inbreeding coefficient based on ROH (F_ROH_), was defined as the proportion of the autosomal genome covered by ROH segments. ROH lengths were taken directly from the PLINK output. Both F_GRM_ and F_ROH_ values were compiled into a single table using individual IDs.

To evaluate the relationship between F_GRM_ and F_ROH_, Pearson’s correlation coefficient was calculated using the cor.test function from the stats package in RStudio (v2024.12.1+563), separately for each population and for the full dataset (n = 497). The analysis was repeated using four minimum ROH length thresholds: 1, 4, 8, and 16 Mb. Results are presented as tables and scatter plots with regression lines and 95% confidence intervals in RStudio (v2024.12.1+563) using the ggplot2 package.

### 2.5. Gene Annotation and Functional Enrichment Analysis

Gene annotation for SNPs in the top 1% on each chromosome was performed using the BioMart platform (version: Ensembl release 114) based on the ARS-UI_Ramb_v2.0 reference genome. Subsequent annotation in UniProt database (https://www.uniprot.org, accessed on 21 August 2025) allowed accurate classification of the candidate genes as proteins. To gain deeper biological insight, we then used the DAVID databases (https://david.ncifcrf.gov, accessed on 21 August 2025) to detect the Kyoto Encyclopedia of Genes and Genomes (KEGG) pathways and Gene Ontology (GO) terms.

## 3. Results

### 3.1. ROH Analysis Results

A total of 41,728 ROH segments were identified across the 497 sheep included in the dataset, with an average segment length of 1588.5 kb. The number and mean length of ROHs varied between populations, ranging from 5656 segments in Alakol-Agro to 8374 in the Nur population. The longest average ROH length was observed in Razakhun (1734.6 mb), while the shortest was in Alakol-Agro (1380.9 mb). A summary of ROH counts and average lengths per population is presented in Table 1.

ROH segments were further classified by length into five categories: 1–2 Mb, 2–4 Mb, 4–8 Mb, 8–16 Mb, and >16 Mb. Table 2 shows the average number of ROHs per length category for each sheep population. Across all populations, the majority of ROH segments were short, predominantly within the 1–2 Mb range, with a gradual decrease in the number of segments as length increased. Segments longer than 16 Mb were rare or absent in most groups.

**Figure 2:**
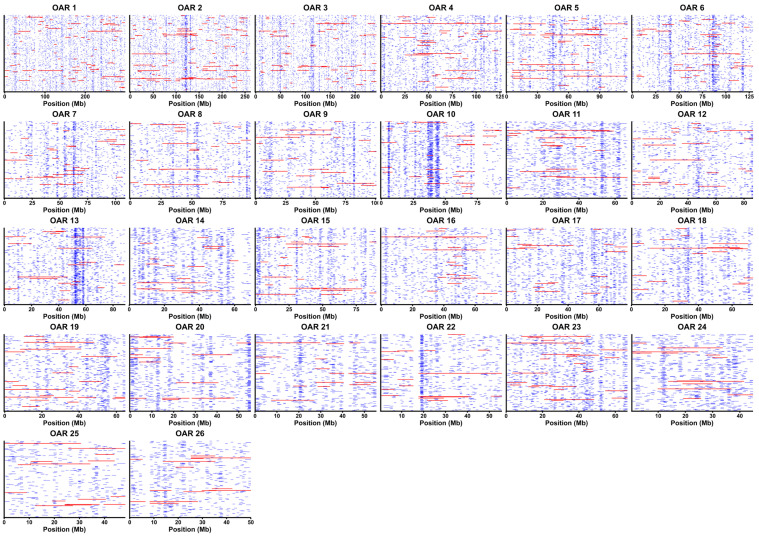
Chromosomal distribution of ROH segments across all autosomes. Horizontal bars represent individual ROHs. Segments shorter than 5 Mb are shown in blue; segments longer than 5 Mb are shown in red.

The chromosomal distribution and variation in ROH length are shown in Figure 2. ROH segments were unevenly distributed across the autosomes, with notable clusters on OAR10 (30–45 Mb), OAR13 (50–70 Mb), and OAR22 (20 Mb), reflecting shared homozygous regions among individuals.

**Figure 3:**
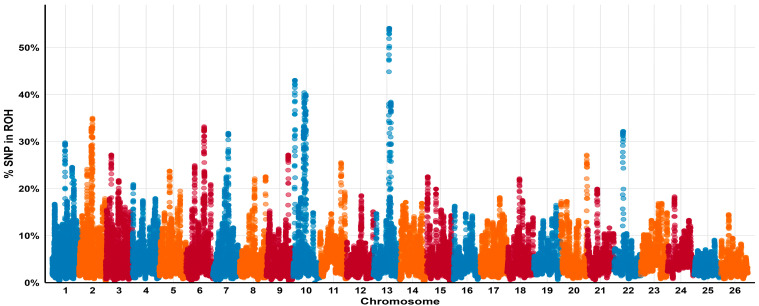
Manhattan plot showing SNP frequency within ROH across 26 autosomes in Kazakh fat-tailed coarse-wool sheep. The x-axis represents chromosome number and SNP position; the y-axis shows the proportion of individuals in which each SNP falls within ROH. The dashed line marks the 99.5th percentile threshold. Clusters above this level are seen on chromosomes 10 and 13, indicating regions of elevated homozygosity.

**Figure 4:**
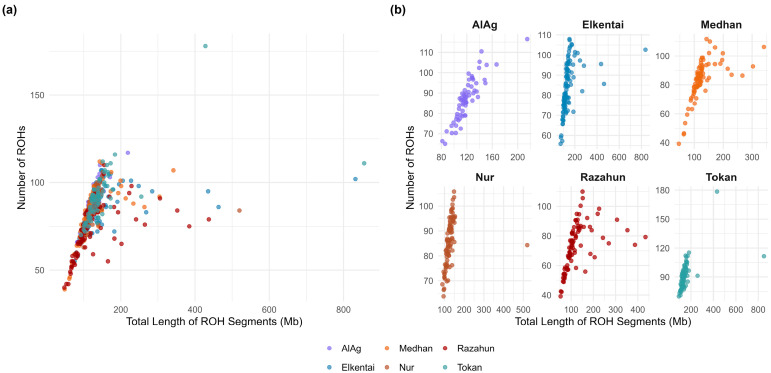
ROH distribution in Kazakh fat-tailed coarse-wool sheep: (**a**) Scatter plot showing the number of ROH segments (y-axis) and their total length in Mb (x-axis) for all 497 individuals. Most animals fall within 40–120 ROH segments and 100–300 Mb, although some exceed 180 segments and 800 Mb. (**b**) The same plot faceted by population (AlAg, Elkentai, Medhan, Nur, Razahun, Tokan) for within-group comparison of ROH count and total length.

To further characterize homozygosity at the SNP level, we assessed the frequency of individual SNPs occurring within ROH regions across the genome (Figure 3). ROH count and total ROH length also varied across individuals and populations, as shown in Figure 4.

### 3.2. Genomic Inbreeding Estimates and Correlation Between F_ROH_ and F_GRM_

Genomic inbreeding levels were assessed using two metrics: F_ROH_, calculated as the proportion of the autosomal genome covered by ROH segments, and F_GRM_, derived from the genomic relationship matrix. The average values of F_ROH_ by length class and the mean F_GRM_ with 95% confidence intervals for each population are presented in Table 3. Genomic inbreeding coefficients varied among populations, with total F_ROH_ ranging from 0.046 in AlAg to 0.058 in Tokan. Mean F_GRM_ values were broadly consistent with F_ROH_, although their wide confidence intervals reflected sampling limitations and estimation variability.

Pearson’s correlation coefficients between individual F_ROH_ and F_GRM_ values were calculated for four ROH length thresholds (>1 Mb, >4 Mb, >8 Mb, and >16 Mb). The results are shown in Table 4. Correlations were consistently high within populations, ranging from 0.58 in AlAg to 0.98 in Tokan. Across the full dataset (n = 497), the correlation between F_ROH_ and F_GRM_ was *r* = 0.58 (t = 15.91, df = 495, *p* < 2.2 × 10^−^^16^; 95% CI: 0.52–0.64), indicating a moderately strong and statistically significant association.

**Figure 5:**
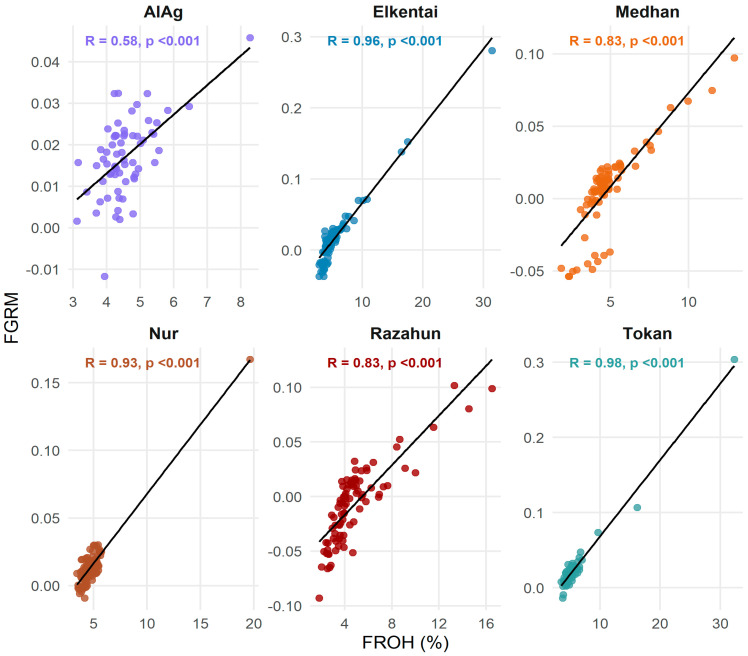
Correlation between F_ROH_ and F_GRM_ within each population. Each panel shows a scatter plot of individual F_ROH_ (x-axis) and F_GRM_ (y-axis) values. Regression lines and Pearson’s R coefficients with *p*-values are indicated.

Despite differences in methodology, F_ROH_ reflecting genomic regions in a homozygous state over extended stretches and F_GRM_ capturing genome-wide allelic similarity, both metrics showed substantial agreement. Their relationship is visualized in Figure 5, where scatter plots with regression lines and correlation statistics are shown for each population.

**Figure 6:**
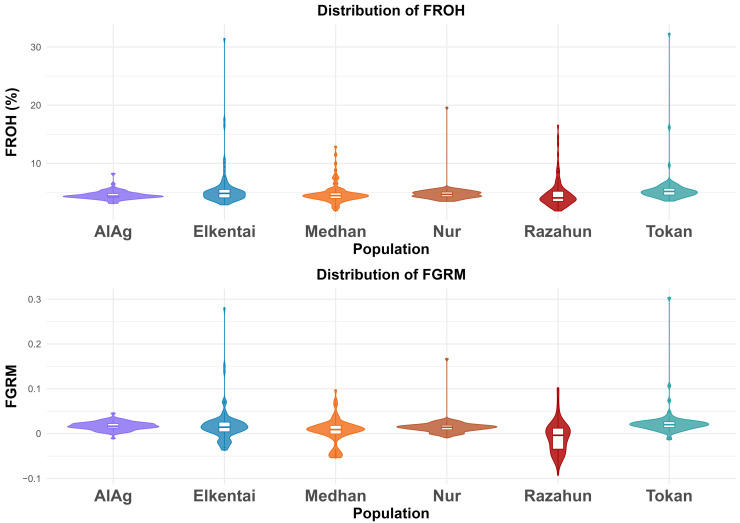
Distribution of F_ROH_ and F_GRM_ across populations. Violin plots show the distribution of genomic inbreeding coefficients by population. The upper panel displays F_ROH_, the lower panel shows F_GRM_.

Moreover, Figure 6 displays violin plots illustrating the distribution of F_ROH_ and F_GRM_ across populations. F_ROH_ values were highest in Tokan, Elkentai, and Medhan, suggesting elevated levels of recent inbreeding. AlAg showed the lowest F_ROH_ levels. The distribution of F_GRM_ values was more concentrated around zero and less variable, consistent with its sensitivity to total homozygosity regardless of segment length. Together, these results indicate that both F_ROH_ and F_GRM_ are informative and complementary in capturing different aspects of genomic inbreeding.

### 3.3. Gene Annotation and Functional Clustering

**Figure 7:**
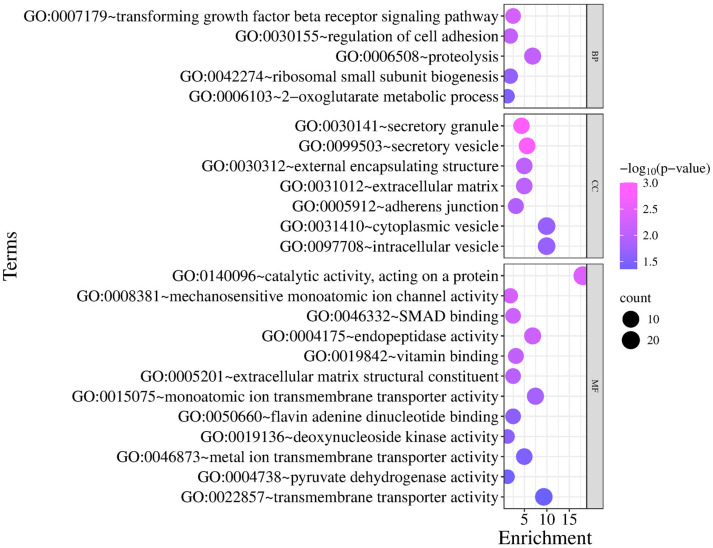
The GO and KEGG pathway enrichment analyses of candidate genes. BP, Biological Process; CC, Cellular Component; MF, Molecular Function. The x-axis represents the enrichment score of each GO term, while the y-axis lists the GO term names. The size of each bubble indicates the number of genes enriched in that GO term (count), and the bubble color corresponds to the statistical significance (−log10 *p*-value).

The GO and KEGG analyses highlighted pathways strongly related to metabolism and growth regulation. The enrichment of transforming growth factor beta signaling (TGF-β), extracellular matrix regulation, metabolic processes, and nutrient transport reflects the complex interplay of pathways underlying sheep productive traits (Figure 7). These results suggest that genes involved in muscle development, energy metabolism, and structural tissue integrity are central to growth performance, carcass quality, and potentially fat deposition in sheep. The identification of arginine, proline, and TCA cycle-related pathways further emphasizes the importance of efficient nutrient metabolism in determining productivity traits (Tables S1 and S2).

## 4. Discussion

Sheep play an important role in the lives of people in Kazakhstan, serving as a vital source of livestock products and cultural heritage. Regular monitoring of genomic diversity and inbreeding is essential for the sustainable development of the breed and the preservation of its adaptive traits. This is the first study to identify ROH in Kazakh fat-tailed coarse-wool sheep.

Analysis of extended runs of homozygosity revealed clusters of long ROHs on chromosomes OAR10, OAR13, and OAR22. In contrast, short and medium-sized ROHs were predominant in other genomic regions and were evenly distributed across the genome, consistent with background recombination. Genomic inbreeding coefficients (Table 3) showed clear population differences. Total F_ROH_ was lowest in AlAg (0.046) and highest in Tokan (0.058), with Elkentai (0.056) also displaying elevated values. The distribution of ROH length classes reflected demographic history: short segments (1–4 Mb) predominated in all groups, indicating the contribution of distant ancestral inbreeding, whereas the presence of long segments (>16 Mb) in Elkentai and Tokan reflected recent consanguinity.

Mean F_GRM_ values were low (from −0.007 in Razahun to 0.025 in Tokan), and the wide confidence intervals indicated estimation variability in smaller populations. The difference between F_ROH_ and F_GRM_ underlined the complementary nature of the two indicators, with F_ROH_ providing the most consistent measure of realized autozygosity.

From a breeding standpoint, the higher levels of genomic inbreeding in Tokan and Elkentai require careful monitoring to prevent further increases, while the comparatively low inbreeding in AlAg demonstrates better retention of genetic diversity. Practical management should include genomic control of mating schemes, avoidance of close-relative pairings, and the use of unrelated sires to maintain effective population size. These measures will help balance genetic improvement with the preservation of genetic diversity, supporting sustainable development of the studied sheep populations. In Iranian fat-tailed sheep, notable ROH islands were detected on chromosomes OAR2, OAR6, and OAR10 [26]. In our previous study, we also observed a high prevalence of short genomic segments in Kazakh meat–wool sheep [18]. Likewise, the majority of ROH segments in Moroccan sheep ranged between 1 and 6 Mb [27]. In addition, among the examined Chinese sheep breeds, Large-tailed Han sheep displayed the highest level of ROH-based inbreeding, while Hulun Buir sheep exhibited the lowest [28]. The contribution of long ROHs (>8 Mb), typically associated with recent inbreeding, was relatively small. However, variation in their abundance between flocks may reflect differences in demographic history and selection pressure.

The strong correlation between F_ROH_ and F_GRM_ (*r* = 0.58) confirms that F_ROH_ is a reliable indicator of genomic inbreeding, especially in populations with limited genetic diversity. The highest correlations were observed in Tokan, Elkentai, and Nur, indicating high levels of inbreeding and reduced genetic variability. In contrast, moderate correlations in Medhan and Razahun may reflect the influence of additional factors such as gene flow or admixture. The lowest correlation was found in AlAg, suggesting greater genetic variability and weaker association between homozygosity and relatedness. Notably, different genomic inbreeding coefficients indicated low levels of inbreeding within the Hu sheep population [29]. A previous study reported that inbreeding coefficients (F_GRM_ and F_ROH_) indicated higher levels of inbreeding and reduced genetic diversity in Iranian wild sheep and goats compared with their domestic counterparts, highlighting the impact of domestication and management practices in maintaining greater genetic variability in farmed populations [30].

An ROH study in global sheep populations has identified several genes associated with growth, body weight, and meat quality traits [31]. In this study, among the genes associated with productivity traits, particular attention is given to the transcription factors myogenic factor 5 (*MYF5*), which play a central role in myogenesis. These genes have been identified as important markers of meat performance in livestock. In Chinese Qinchuan cattle, polymorphisms in *MYF5* are associated with growth and carcass quality [32], while in goats, expression variability in both genes has been linked to muscle fiber morphology, including cross-sectional area and density [33]. In dairy cattle (e.g., Brown Swiss and Holstein), *MYF5* alleles have shown associations with feed efficiency [34], indicating the broader relevance of these markers beyond beef breeds. PR/SET domain 16 (*PRDM16*) is a key regulator of lineage determination between brown adipose tissue and skeletal muscle. Studies have shown that *PRDM16* expression in myoblasts promotes brown adipogenesis via interaction with transcription factors such as PPARγ and C/EBPβ, while its inactivation in brown fat precursors induces expression of myogenic markers (MyoD, Myogenin) and leads to muscle formation in MYF5-positive lineages [35]. These mechanisms are of practical interest in animal production, as the balance between fat and muscle affects carcass composition and meat quality. According to Lee and Hong (2024), the solute carrier family 26 member 4 (*SLC26A4*), which encodes anion transporters, is involved in regulating intracellular pH via Cl^−^/HCO_3_^−^ exchange [36]. Changes in acid–base balance may indirectly influence lipogenesis, including the expression of key lipogenic enzymes. This regulation may affect fat deposition and meat marbling.

The transglutaminase 3 (*TGM3*) gene, which encodes transglutaminase 3, was identified among genes associated with wool quality. John et al. (2012) reported that knockout of Tgm3 in mice resulted in severe hair shaft defects, including cuticle deformation and reduced tensile strength, despite an intact epidermal barrier [37]. These effects were attributed to disrupted cross-linking between trichohyalin and keratins in the absence of TGase 3 activity. As keratin matrix stability underpins wool structure, *TGM3* might be a functionally relevant candidate for improving fleece characteristics in sheep breeding programs.

SMAD5 is a receptor-regulated SMAD (R-SMAD) activated by bone morphogenetic proteins (BMPs) within the TGF-β signaling pathway. It plays a crucial role in processes directly linked to productive traits in sheep [38]. A previous study reported *SMAD5* expression in Sertoli and Leydig cells during various developmental stages of the Tibetan sheep testis [39]. In addition, RNA-sequencing of ovine ovary tissue revealed that this gene might play a role in the actions of estrogen [40].

Collectively, the revealed genes may serve as valuable targets for marker-assisted selection to improve muscle development and wool traits in Kazakh fat-tailed coarse-wool sheep and warrant further investigation to clarify their functional roles.

## 5. Conclusions

This study revealed patterns of genetic diversity and inbreeding in Kazakh fat-tailed coarse-wool sheep. The most pronounced ROH were located on chromosomes OAR10, OAR13, and OAR22, potentially reflecting signatures of selection. Across populations, homozygosity was predominantly concentrated in short segments (1–4 Mb). A strong positive correlation was observed between the genomic inbreeding coefficients F_ROH_ and F_GRM_. Furthermore, several functionally important genes (*MYF5*, *PRDM16*, *TGM3*, *SLC26A4*, *SMAD5*) were identified within these regions, which play roles in productivity traits. Taken together, these findings provide new insights into the molecular mechanisms underlying productivity, and they offer a foundation for more accurate genetic evaluation and the implementation of marker-assisted selection programs in this breed.

## Figures and Tables

**Table 1:** Number and average length of ROH segments in each population.

Population	Total Number of ROH (n)	Mean ROH Length (mb)
AlAg	5656	1.381
Elkentai	8267	1.721
Medhan	6613	1.530
Nur	8374	1.490
Razahun	6321	1.735
Tokan-1	6497	1.645
Total	41,728	1.589

Note: values rounded to one decimal point for clarity.

**Table 2:** Distribution of ROH segments by length classes across six sheep populations.

FID	ROH_1-2 Mb	ROH_2-4 Mb	ROH_4-8 Mb	ROH_8-16 Mb	ROH_>16 Mb	Total ROH
AlAg	81 ± 9.3	5.4 ± 2.6	1.5 ± 0.9	1.7 ± 1.5	NA	87 ± 10.2
Elkentai	75.7 ± 11	6 ± 2.8	2.2 ± 1.7	2.3 ± 1.9	3 ± 3.9	84.4 ± 12.2
Medhan	77 ± 12.3	5.2 ± 2.2	2.3 ± 2.2	2.4 ± 2.1	1.9 ± 1.2	83.7 ± 13.6
Nur	78.1 ± 8.7	5.9 ± 2.4	1.6 ± 0.8	1.3 ± 1.2	3 ± 4	85.4 ± 9.2
Razahun	66.2 ± 13.7	5 ± 2.8	2.3 ± 1.6	2.4 ± 1.9	3.2 ± 2.6	73.5 ± 14.9
Tokan	81.9 ± 10.3	6.8 ± 4.5	2.4 ± 3.1	1.7 ± 1.5	2.7 ± 4.3	91.5 ± 14.8

Note: NA, not available; values are presented as mean ± SD.

**Table 3:** Genomic inbreeding coefficients in sheep populations.

Population	F_ROH_ 1–4 Mb	F_ROH_ 4–8 Mb	F_ROH_ 8–16 Mb	F_ROH_ >16 Mb	Total	F_ROH_-F_GRM_ (Mean ± CI)
AlAg	0.045	0.001	0.001	0.000	0.046	0.017 (−0.012–0.046)
Elkentai	0.043	0.003	0.003	0.007	0.056	0.017 (−0.037–0.281)
Medhan	0.042	0.002	0.002	0.003	0.049	0.006 (−0.054–0.097)
Nur	0.044	0.002	0.001	0.002	0.049	0.015 (−0.009–0.167)
Razahun	0.037	0.002	0.004	0.006	0.049	−0.007 (−0.093–0.102)
Tokan	0.047	0.004	0.002	0.005	0.058	0.025 (−0.014–0.304)

Note: Average F_ROH_ values by ROH length class and total F_ROH_ are shown for each population. Mean F_GRM_ values with 95% confidence intervals are also included.

**Table 4:** Pearson correlation coefficients between F_ROH_ and F_GRM_ at different minimum ROH length thresholds (>1 Mb, >4 Mb, >8 Mb, >16 Mb) across sheep populations.

Population	*r* (>1 Mb)	*r* (>4 Mb)	*r* (>8 Mb)	*r* (>16 Mb)	*r* (Total)
AlAg	0.451	0.397	0.486	NA	0.583
Elkentai	0.233	0.698	0.706	0.894	0.959
Medhan	0.576	0.483	0.554	0.649	0.831
Nur	0.103	0.181	0.819	0.878	0.932
Razahun	0.604	0.639	0.488	0.654	0.832
Tokan	0.361	0.514	0.752	0.918	0.980

## Data Availability

The data are available upon request.

## Supplementary Materials

The following supporting information can be downloaded at: https://www.mdpi.com/article/10.3390/ani15182714/s1. Table S1: Summary of gene annotations retrieved from UniPorts; Table S2: The KEGG-enriched terms.

## Abbreviations

The following abbreviations are used in this manuscript:

ROH	Runs of homozygosity
FROH	Inbreeding coefficient based on ROH
FGRM	Inbreeding coefficient based on genomic relationship matrix
MAS	Marker-assisted selection
SNP	Single-nucleotide polymorphism
